# The linguistic situation on the Ukrainian Black Sea coast – Ukrainian, Russian and Suržyk as “native language”, “primary code”, frequently used codes and codes of linguistic socialization during childhood

**DOI:** 10.1007/s11185-022-09259-4

**Published:** 2022-09-14

**Authors:** Gerd Hentschel, Olesya Palinska

**Affiliations:** Carl von Ossietzky University, Oldenburg, Germany

## Abstract

The study analyses the linguistic situation in the three Ukrainian oblasts on the Black Sea coast using survey data collected from 1,200 respondents before the Russian attack on Ukraine. At the end of the 18th century, this region was the core of a “new Russian” governate during Tsarist times. Previously, the region had been ruled by Tatars and there were neither Russian nor Ukrainian settlements. From the 19th century onwards, the Ukrainian and Russian population dominated. Since the annexation of the Crimea, these oblasts represent a crucial part of the Kremlin’s plan to establish an “expanded New Russia (Novorossiya)” under Moscow’s control – extending along the Ukrainian-Russian border and the northern Black Sea coast, reaching from Xarkiv to Odesa. This area is clearly at the forefront of Russia’s current war goals since controlling it would allow them to establish the strategically important land bridge to Crimea.

Linguistically, the area undoubtedly belongs to those regions of Ukraine where Russian was prominent, although apart from the Crimea at no time was there an ethnic Russian majority on the Black Sea coast – neither during Soviet times nor since Ukraine’s independence. This means that the population with Ukrainian “nationality” also made strong use of Russian. This situation is being instrumentalized by Moscow as an argument for its military intervention to protect the Russian or Russian-speaking population.

The study firstly describes the linguistic situation in the region, differentiating between the so-called mother tongue, the first language acquired and the principally-used language. It can be shown that the traditionally assumed dominance of Russian is actually far weaker when the population’s “multicodality”, including the mixed variety Suržyk, is included in the analysis. A differentiation is made between respondents with Ukrainian and Russian nationality throughout the analysis. Using statistical procedures such as principal component analysis and cluster analysis, the interdependencies between stated mother tongue, first language and multicodality are presented. Different motives for claiming a certain mother tongue can be identified among subgroups of respondents. The analysis focuses particularly on the questions of the extent to which central government measures to strengthen the position of Ukrainian since Ukraine’s independence have changed respondents’ preferences when choosing a code, and whether respondents have perceived social pressure for any form of shift. On the whole, it can be established that speakers with Ukrainian nationality who were primarily socialized in Russian have considerably increased their usage of Ukrainian, but without abandoning Russian. At best, this can also be established to a minimal extent for respondents with Russian nationality. Furthermore, since there is only extremely scant evidence that respondents encounter disapproval or censure from their environment for their choice of code (be it Russian, Suržyk or Ukrainian), Moscow’s claim of persecution, if not genocide of the Russian-speaking population is exposed as a blatant lie.

## Background

Apart from the Crimean Peninsula, annexed by Russia in 2014, the Ukrainian oblasts on the Northern coast of the Black Sea (including the Sea of Azov), hold a prominent position in the predatory-prey pattern of Russia’s neo-imperial aggression under Putin’s government, of course together with the Donbas area and Xarkiv. The Black Sea oblasts would guarantee continental access to the Crimean naval port of Sevastopol, which is of major importance for Russian imperial interests – not only in the Black Sea, including the so-called Balkan area with the Russia-oriented Transnistrian pseudo-state, but also in the Mediterranean area as well as Africa and all of the Middle East. Historically, the territory of the contemporary Ukrainian Black Sea oblasts formed the core of so-called “Novorossiya”, literally New Russia, or, to be precise, the Novorossiya Governate of the Russian Empire established during the second half of the 18th century. In the first half of the last millennium, these territories did not have an autochthonous Slavic population, neither Ukrainian (or “Little Russian” in the terminology of the Russian Empire) nor Russian (“Great Russian” in the same terminology). Serfs fleeing from more central parts of the Polish-Lithuanian Commonwealth started to settle in these less controlled areas called “Wild Fields” in the 15th century. There, over the course of time, the Zaporizhian Sich and the Cossack Hetmanate developed, a semi-autonomous polity, which came to an ultimate end by 1775. The following colonization of these territories in Tsarist times was multi-ethnic, with Ukrainians and Russians being the largest groups in the long run, with differences between the share of the two groups over time, regions, cities / towns and villages. By the end of the 19th century, it suffices here to state that the All-Russian Empire Census (1897) provides evidence for a linguistic constellation in which most of the population spoke Ukrainian, whereas in cities Russian and “Jewish” (which meant Yiddish) dominated clearly. Thus, by the end of the 19th century, Ukrainian was mainly the language of the countryside.

The historical background,^1^ i.e. the fact that the conquest of the Black Sea territories by the Russian Empire was taken as a motivation for Putin’s enlarged “Novorossiya project” of 2014 (cf. Bidder, 2014; Basora & Fischer, 2014,2), gained renewed interest in the context of the annexation of the Crimean Peninsula. The envisaged federal state should have included the oblasts of Xarkiv, Donec’k, Luhans’k, Zaporižžja, Xerson, Dnipro (Dnipropetrovs’ka oblast), Mykolaïv, Odesa, and Crimea, with a total population of approximately 21 million. Although these plans somehow faded into the background over the following years, it is obvious that it is precisely this area that has been the focus of the attacks by the Russian military forces since March 2022, after the fruitless endeavours to capture the Ukrainian capital of Kyïv from the North.

Apart from the Crimea, in all oblasts mentioned, ethnic Russians were in the minority, Ukrainians in the majority. This was the case in the first and until today only census taken in independent Ukraine in 2001 as well as in the last census taken by the Soviet Union in 1989 (for specific figures cf. Ukrainian Ukrainian Census, 2001 for both sources). Between 1989 and 2001, there was a decrease in the share of the Russian population in single oblasts of between roughly 5 and 7 percentage points and a corresponding increase in the share of the Ukrainian population. To what extent these changes are based on migration (e.g. emigration of Russians from Ukraine to the Russian Federation) or on a redefinition of ethnicity cannot be discerned here and is of minor importance due to the subtlety of quantitative shifts. It is more than plausible that this development has continued up to today, be it also due to the migration of Ukrainians from the oblasts of Luhans’k and Donec’k because of the war-like situation there since 2014.

**Map 1:**
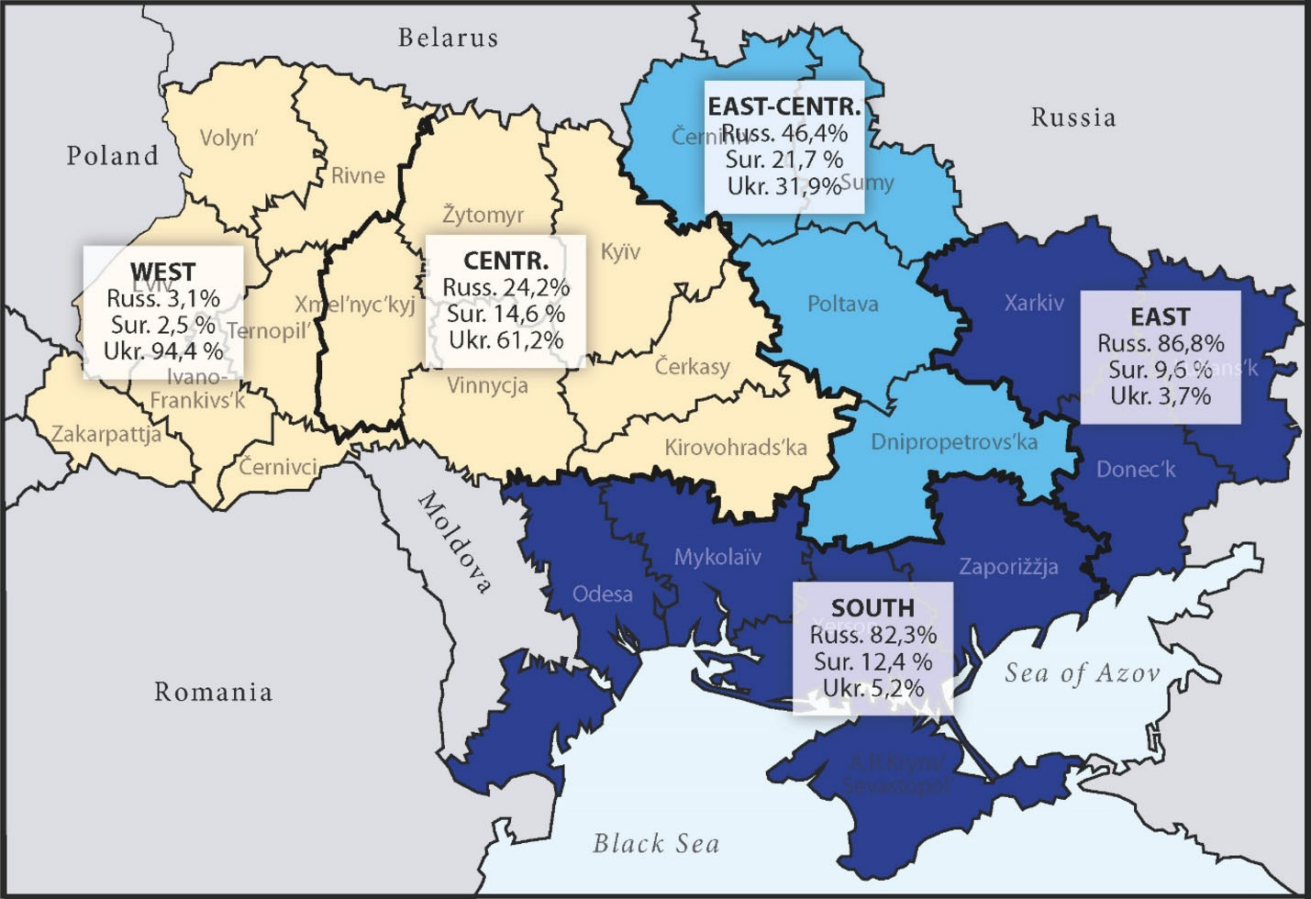
The distribution of the three codes according to principal usage (according to KIIS, 2003). Source: reproduced from Hentschel and Taranenko (2021)

As a matter of fact, the linguistic distribution of Ukrainian and Russian in the area of interest differs from the shares of the ethnic groups in the sense that Russian is considered to be spoken far more often than Ukrainian (cf. for example Kulyk, 2014). This of course means that by far not all of those who use mainly Russian are ethnic Russians. The area envisaged for a federal state of Novorossiya largely corresponds to the one for which even the Kiev International Institute for Sociology (KIIS) stated a clear, absolute dominance of Russian as “principally used code” over Ukrainian (KIIS,^3^,^2003^) – the areas marked by (dark) blue in Map 1.

In this KIIS analysis a third code, in addition to Ukrainian and Russian, is also illustrated: Suržyk.^4^ Roughly speaking, this is a mixed Ukrainian-Russian variety whose grammatical and phonic (phonetic and phonological) phenomena are on the whole more Ukrainian-like than Russian-like. The lexicon however shows a considerable influence from Russian.^5^ Of course, Suržyk has a completely different status than Ukrainian and Russian. It is an oral vernacular in which the Ukrainian “element” is partly shaped by old rural dialects, partly by the standard language; and the Russian “element” rather by Standard Russian (cf. Hentschel & Palinska, 2022). Due to the colonial-like constellation of Russian as the socially and politically dominating language,^6^ and Ukrainian as the language of the dominated autochthonous people, Suržyk is viewed rather negatively by Ukrainian elites (cf. Stavyc’ka, 2014), although slight changes to the positive have been observed (cf. Bilaniuk, 2018). Among the population of (Central) Ukraine, the attitudes towards the mixed code range from clear rejection to clear approval (cf. Hentschel & Zeller, 2016) and the quantitative relation between respondents rejecting or approving of Suržyk is relatively balanced.

All but one oblast that Kremlin plans designate as future members of the Novorossiya state are part of those regions of the South and the East for which the KIIS map above states an absolute predominance of Russian with a share of more than 80 percent. Only the oblast of Dnipro belongs, in terms of the KIIS (2003) classification, to the East of Central Ukraine, for which a relative predominance of Russian was stated: on average somewhat less than 50 percent.

**Map 2:**
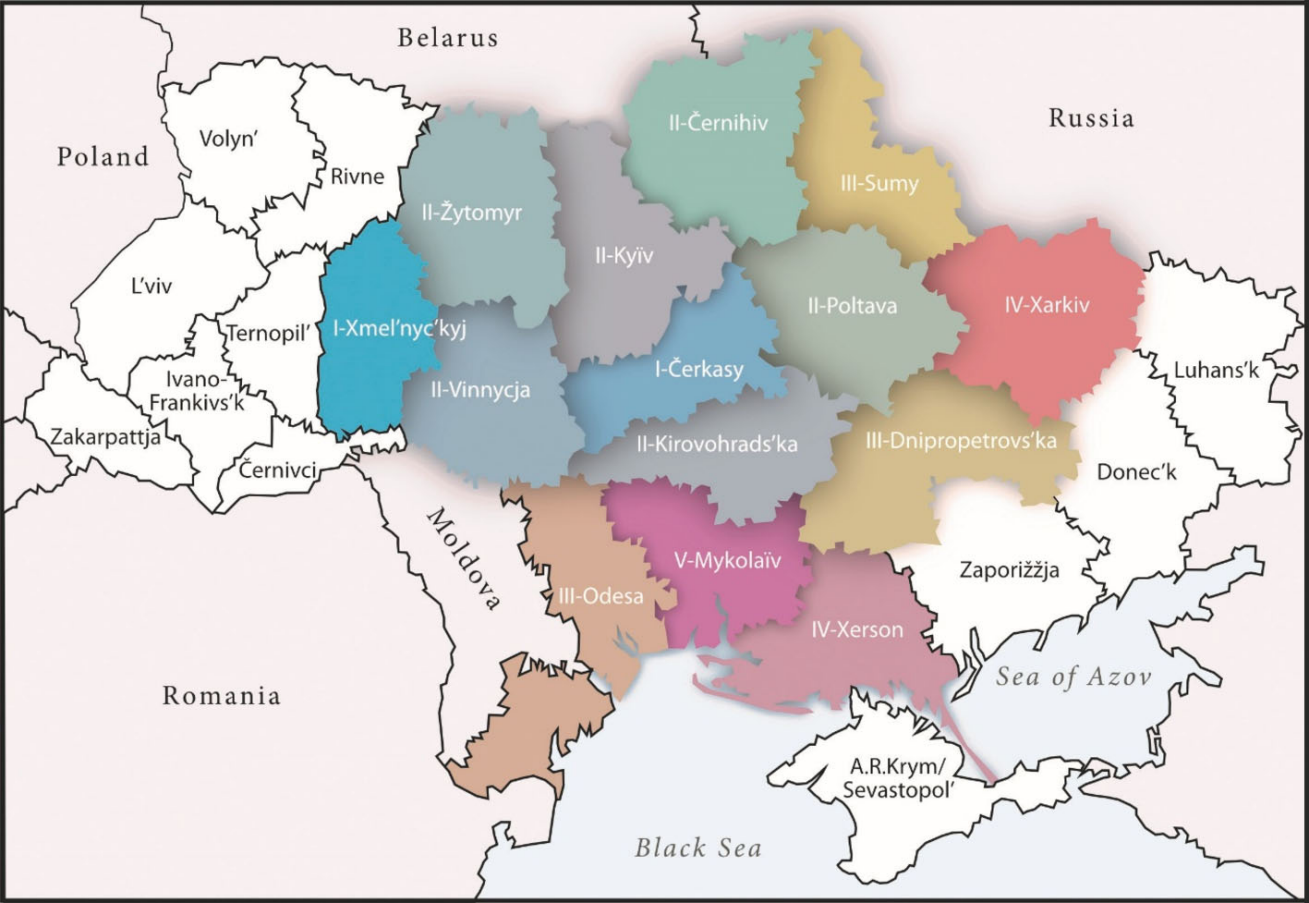
The strength of the three codes in the oblasts – clusters indicated by Roman numerals. Source: reproduced from Hentschel and Taranenko (2021)

The KIIS study has the disadvantage that it concentrates on the principally or mainly used code, thus ignoring the very widespread multilingual or multicodal linguistic practice in the population. Furthermore, by averaging the figures for larger regions such as South, East, Eastern Centre etc., which are roughly based on historical regions of Ukraine, a bipartite Ukrainian-Russian division of Ukraine is implied (cf. the contrast between blue and ochre in Map 1). To overcome these and other insufficiencies, Hentschel and Taranenko (2021) developed a different approach.^7^ In surveys for two research projects^8^ on Suržyk, concentrating on Central Ukraine or the three Black Sea oblasts of Odesa, Mykolaïv and Xerson, 1,400 and 1,200 respondents respectively were asked in 2014 and 2019/2020 respectively, how often they use each code in everyday life. The possible answers were graded in five steps from ‘never’ to ‘all the time’. Then, an index was calculated that fixed the presence of the codes for each oblast by considering the data from each respondent. The composition of the colouring^9^ of the oblasts is directly based on the index for the codes in each oblast. Thus, practically every oblast has an at least slightly different colour to all others. Nevertheless, five clusters of oblasts, each with relatively similar quantitative relations in the usage of the three codes remain visible (cf. Map 2 below). The borders of the oblasts simply serve as a kind of coordinate system within which average figures have been calculated. Differences within single oblasts and thus potentially smooth transitions in linguistic practice, which are definitely there to a certain degree, could of course not be modelled. Nevertheless, Map 2 illustrates that linguistic practice in the parts of Ukraine that could be considered^10^ are much more gradual in character than presentations of the kind published by KIIS (2003) suggest.

In Map 2, a colouring towards blue indicates the strength of Ukrainian, towards red the strength of Russian, and towards yellow of Suržyk. The five abovementioned clusters of oblasts are indicated by Roman numerals preceding the name of the oblasts:

In the following, we will concentrate on the three Ukrainian oblasts on the Black Sea coast in the narrow sense, i.e. without Zaporižžja and Donec’k on the Sea of Azov, which are the focus of interest in the still ongoing research project mentioned above (cf. Hentschel & Reuther, 2020). As can be seen from the colouring of the three oblasts, Odesa is much less Russian oriented than Mykolaïv and Xerson.^11^

For the three oblasts at issue here, the main figures of interest underlying the colourings are as follows: In the oblasts of Mykalaïv and Xerson, it is Russian that dominates the linguistic situation in everyday life. In Mykolaïv this predominance is absolute, with 62 out of 100 possible index points. In Xerson we find a relative predominance with 47 points. In Odesa (as already mentioned, only the oblast, without the city^12^) Russian receives a few less index points than Suržyk: 36 vs. 40. Thus, Suržyk is relatively predominant in the oblast of Odesa, but less present in Xerson, 26 points, and much less so in Mykolaïv, only 12 points. The share of Ukrainian is relatively stable in all three oblasts, about 25 points. Compared^13^ with the figures from the analysis by KIIS (2003), one can state that the results for Russian are much lower, although Russian is still predominant. The main reason for this is probably that not only the principally used code was asked for (cf. Hentschel & Taranenko, 2021 for more details). Our data show that only one in ten respondents report that they never use Ukrainian. The same share holds for Russian. And three in ten state that they never use Suržyk. By including the usage of codes other than the mainly used one into a quantifying description of the presence of all codes, the share of the mainly used one is automatically reduced. Furthermore, the KIIS (2003) analysis was conducted almost 20 years before our survey, and there can be no doubt that Ukrainian now takes up more space in the population’s linguistic practice and that the reluctance to admit to practising Suržyk has declined as well. We will return to this topic in the analytic part of the paper.

In what follows, we will offer a first comprehensive account of the linguistic situation in the three oblasts of Odesa, Mykolaïv and Xerson, which are the focus of the abovementioned research project on the Ukrainian Black Sea coast. The paper is thus an initial report on ongoing research in the area – one of the genres the editors of this special volume on Ukraine wanted to include. The first point that will be at issue is the question of native language (or “mother tongue”, Ukr. “ridna mova”, Russ, “rodnoj jazyk”) in a society like Ukraine. Several conceptions of native language will be highlighted and related to the three codes. Second, information on respondents’ “primary code” will be provided, accompanied by a discussion of the methodological problems confronting quantitative analyses based on this concept in multilingual societies. Third, the contemporary linguistic practice or current usage of the three codes will be discussed and, subsequently, the code of linguistic socialization during childhood will be investigated. Last but not least, the question of shifts between the codes over people’s lifetimes will be posed.

## Native language

### Native languages and ethnic groups (“nationality”)

Language usage in the sense of acknowledged languages or other codes, e.g. vernaculars, has an impact on the statement of one’s native language or “mother tongue”. But this statement has at least partially a different character because it can be only loosely, sometimes more, sometimes less connected with respondents’ actual linguistic practice,^14^ at least in multilingual societies. Such a statement is often an implicit utterance of attitudes towards the languages or other codes at issue, in which various factors play a role. This has been widely discussed in the literature (cf., for example, Skutnabb-Kangas & Phillipson, 1989; O’Rourke & Ramallo, 2011). Zeller (2021) has offered a first multifactor analysis for concepts of native languages in Central Ukraine, which is based on material from the second project mentioned above and thus concentrates on the oblasts that have been coloured in Map 2, apart from Odesa, Mykolaïv and Xerson. We will refer to his study at various points in what follows.^15^

**Fig. 1:**
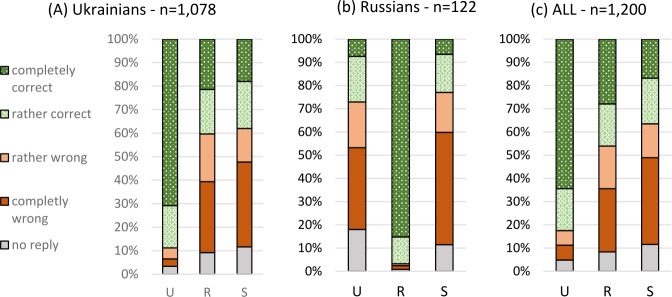
The three codes as native languages (mother tongues)

The immediate question for the situation in Ukraine, not only in the three oblasts at issue here, is that of the ethnic groups of Ukrainians and Russians. We will start our analysis with this aspect. The results are presented in Fig. 1, where we differentiate speakers who state that they belong to the Ukrainian or Russian ethnic group^16^ in Fig. 1a and 1b:

One difference between Ukrainian and Russian respondents is obvious. The latter declare Russian, their “titular language”, to be their native language with only very few exceptions, although every tenth respondent only reservedly agreed with the statement. Ukrainians do so with Ukrainian to an extent that is only a little lower, but every tenth respondent rejects Ukrainian as their mother tongue. The attitudes to the respective other language, i.e. Ukrainian or Russian, as well as to Suržyk are similar for both ethnic Ukrainians and Russians, although the acknowledgement of the other two codes among Ukrainians is a little higher. The acknowledgement of Russian as a mother tongue can be observed for roughly 550 respondents, only one fifth of whom are Russians, the others Ukrainians. Among the latter, three in four exhibit a reserved acknowledgement of Russian as their native language.

The number of Russians among the respondents is thus comparatively small (but at least approximately corresponds to the share of Russian in the population of the region)^17^ and thus has a restricted influence on the whole population of our respondents, as is visible in Fig. (1c). This is much more similar to (1a) than to (1b). In what follows, we will consider all respondents as a rule. The analyses often compare subgroups of respondents, which is not possible for the Russian group separately, due to their small number. Specific observations for this group will be presented when the quantitative basis is solid.

After 2013 / 2014, i.e. after the Russian annexation of the Crimean Peninsula and in the light of the military conflict between the Russian Federation and independent Ukraine in the Ukrainian oblasts of Luhans’k and Donec’k, and in still other words, in times of severe pressure from the Russian Federation on Ukraine, it is far from surprising that many Ukrainians feel more Ukrainian than previously. Quite a number of Ukrainians (here in the sense of Ukrainian citizens of “Ukrainian nationality”) who mostly used Russian as their principal medium of communication before the cited conflicts or even in Soviet times, state that Putin has finally made them convinced Ukrainians. Accordingly, given the background of such a social atmosphere, it is not surprising that even on the Black Sea coast, which most earlier investigations described as overwhelmingly Russian speaking (cf. the discussion in Hentschel & Taranenko, 2021), it is Ukrainian that receives the clearly highest number of mentions as the mother tongue: Slightly over 90 percent of ethnic Ukrainians agreed that Ukrainian was their native language, the overwhelming majority (two thirds of all respondents questioned) with absolute agreement. It should be noted here that in the last Soviet census and in the last and only Ukrainian census only a little more than half of the ethnic Ukrainians named Ukrainian as their native language. For Russian, the rate of agreement today is much lower than for Ukrainian: about 40 percent, with a certain preponderance of absolute agreement (somewhat more than one quarter of all respondents) over a reserved one. More than one third of the respondents even agreed with the statement that Suržyk was their native language, with slightly more reserved agreements than absolute ones.

At this point it must be underlined that there are many cases of multiple statements of native languages. Taking into consideration only those 977 respondents who provided an answer (positive or negative) for all three codes (missing responses are hard to interpret in this respect), then we can state the following: Only roughly 40 percent stated that they have just one native language: 30 percent Ukrainian, 10 percent Russian and less than 1 percent Suržyk. Another roughly 40 percent stated two native languages: 20 percent Ukrainian and Suržyk, 17 percent Ukrainian and Russian, 2 percent Russian and Suržyk. About 17 percent stated that all three codes are their native languages.

A brief comment on reservedly rejecting one or the other codes: If these reserved rejections were to be accepted as some, although clearly restricted form of “felt mothertongueness”, the percentage of Suržyk and Russian would increase considerably, the former reaching about 50 percent the latter about 66 percent. Ukrainian (already on a high level) would gain much less: close to 90 percent rather than a little more than 80 percent.

### Laymen’s conceptions of native language

Declaring a language, a variety, or a code – to use the most neutral expression – to be one’s native language or “mother tongue” has, as has been mentioned above, at least to some degree a symbolic, attitudinal character, expressing identity, loyalty and the like. On the basis of a multifactorial approach it should thus be possible to grade the attachment to single codes. This, of course, holds especially when there is a “choice” between two or more codes in multilingual or “multicodal” societies. In this investigation, the respondents were presented with three statements, one for each code: “Ukrainian / Russian / Suržyk^18^ is my native language”. Furthermore, they were asked to evaluate each of the three statements as “absolutely correct, rather correct, rather wrong, or absolutely wrong”. There was also the explicit option not to comment on each statement. In addition, respondents had been informed that the statements were independent from each other, i.e. they were free to state more than one code as their native language, either on the same or a different level.

The notion of “native language” or “mother tongue” is rather a layman’s category and as such it is multi-layered. Trying to grasp some and, hopefully, the most important layers or aspects of the notion, the respondents were asked to evaluate nine statements of the type “For me, the native language is a language, which …”. Here, there were only two options for the evaluations, yes or no. The statements that were evaluated are shown in the column *Statement* in Table 1. (The values to the right of that column will be commented on below).

**Table 1:**
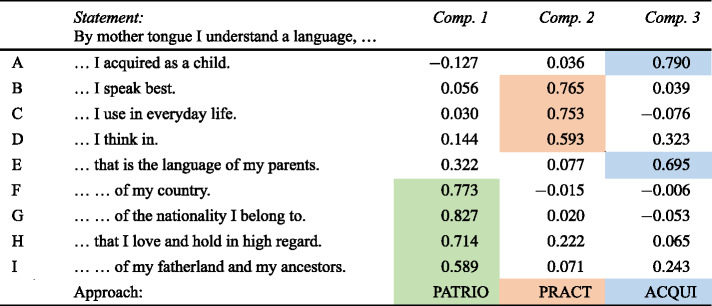
Approaches to a concept of “mother tongue” / native language (Color table online)

There is no need to present descriptive quantitative data on the statements in detail. All but one statement did not reach a majority of positive reactions. This exception was that two thirds of the respondents agreed on point (A), that the native language is a code they acquired during childhood. It should be underlined that this agreement is by no means exclusive. Almost all respondents who agreed with this evaluation also agreed with at least one if not more of the other nine as well, however with large differences. The other statements reached agreement rates of between a minimum of 27 percent (B – the language I speak best as well as H – the language I love and appreciate) and a maximum of 40 percent (E – the language of my parents). The latter, E, and A as the only point agreed upon by the majority are, of course, in a way connected with each other. Both typically refer to the language acquired during childhood, a process in which parents are involved, at least in most cases. Such interdependencies can be stated for other points as well.

In order to base this assumption not only on intuition, a factor component analysis has been calculated.^19^ The results are presented in the three columns on the right-hand side of Table 1 above. The figures presented are coefficients of correlation. In statistics, it is usually assumed that a value of 0.5 or higher signals a correlation of medium strength and a value of 0.7 or higher a strong one. The values in Table 1 represent the strength of the correlation (interdependence) between each of the nine criteria for naming a code the native language on the one hand and the three abstract components on the other hand. As can be seen, between two and four of the nine criteria nicely group together with one of the components by showing correlations of at least medium, but mostly high strength with the corresponding component. These components can now be interpreted:

i. Positive reactions to the statements “By ‘mother tongue’ I understand …” F – “… the language of my country”; G – “… of the nationality, I belong to”; H – “… I love and hold in a regard”; I “… of my fatherland” hint at an emotional, rather patriotic approach (PATRIO) to the notion of “native language” – the first component.
ii. The second component with high values for B – “… the language I speak best”; C – “… I use in everyday life”; D – “… I think in” rather signal a practical approach (PRACT). based on the language(s) or code(s) in their current real linguistic situation.
iii. The third component with high values for A – “… I acquired as a child” and E – “…the language of my parents” is practical or realistic in a similar way to ii. However, it does not refer to the current linguistic practice, but rather to that during childhood. One could speak of an acquisition approach (AQUI) to the notion “native language”.

It should be noted that only the patriotic approach is a symbolic one, mirroring attitudes to a language or code. The practical and the acquisition approach are realistic approaches in the sense that has just been mentioned.

Similar to Hentschel and Zeller’s (2016) investigation of specific components determining linguistic attitudes towards Suržyk, we will now investigate the extent to which these components can be used to group speakers (respondents). In other words: how and to what extent are these components core approaches shared by respondents? It should be noted that, theoretically, the three approaches (components) are not mutually exclusive and respondents may adhere to more than one of them, in the same way as they mostly agreed with more than one of the statements presented.

The statistical method to achieve this aim is cluster analysis. For a high number of elements to be clustered (in our case 1,200 respondents) the adequate variant of cluster analysis is a centroid-based clustering (*k-means* clustering). For a *k-means* cluster analysis the number of clusters has to be fixed in advance. One may either predetermine the clusters and thus their number to be tested by reasonable non-quantitative hypotheses or fix the ideal number of clusters by another variant of cluster analysis: hierarchical cluster analysis. This method can only be run on a smaller number of elements to be clustered, which have to be drawn by random selection from the larger set of elements. As there are no hypotheses or suggestions at hand on how many (and which) groups of respondents should be distinguished on the basis of the three components, the latter approach was taken. The hierarchical cluster analysis was executed on 50 randomly selected respondents and yielded an ideal number of four clusters for the analysis of centroid-clustering that was conducted on the data of all 1,200 respondents. The latter yielded the results illustrated in Table 2.

**Table 2:**
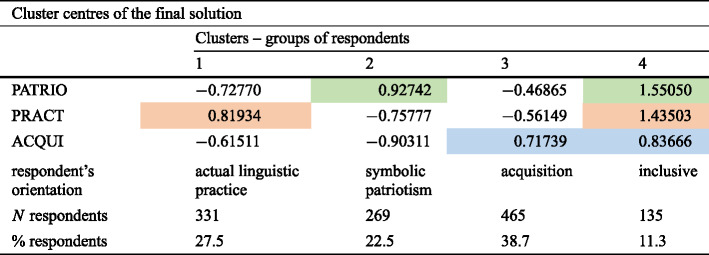
Groups of respondents and different approaches to “mother tongue” (Color table online)

As can easily be seen, the respondents can be almost completely grouped on the basis of the three components, by showing higher positive values for only one of them and comparatively high negative values for the other two. Almost 90 percent of the respondents can be clustered or grouped in this way. Only a comparatively small group of respondents, some 11 percent, show high positive values for all three components, especially for the components PATRIO and PRACT.

On the basis of their approach to the concept of “native language”, the fixed groups of respondents or speakers will from here on be called speakers with an orientation towards

- current linguistic practice (PRACT)
- symbolic patriotism (PATRIO)
- acquisition during childhood (ACQUI)
- an inclusive bundle of all components (INCL)

For the first three, the same abbreviation will be used as for the components, for the last one the new abbreviation “INCL”.

As can be seen in the last two lines of Table 2, the symbolic patriotism motivation for declaring a language to be a native language can only be observed for almost one quarter of the respondents. For a somewhat larger proportion of the respondents, their current linguistic practice and competence plays the dominant role (27.5 percent). The largest group of respondents equates their first language with their mother tongue. In a way, the latter group of speakers understands the term “native language” or “mother tongue” literally (38.7 percent). Last but not least, there is the small group of “inclusive” respondents (11.3 percent). Such a combined approach to a “mother language” can rather be expected in a monolingual society, where at least the components PRACT and ACQUI would not conflict and, if there is some sort of linguistic patriotism in this society at all, it would fit together with the former two components.

Regarding the quantitative data presented in the last lines of Table 2, one very clear difference between Ukrainian and Russian respondents must be underlined: the latter are almost absent from the groups with a symbolic-patriotic or inclusive approach (PATRIO and INCL). For Ukrainians’ understanding of mother tongue on the other hand, the first language acquired is quantitatively more important than the currently most practised code. This point will be returned to below.

The survey we conducted was accompanied by open, in-depth interviews from which we can present utterances made by interviewees which illustrate the abovementioned classification from (a) to (d):^20^

- current linguistic practice (PRACT)
  1. Суржик я счітаю своїм рідним язиком. (...) всі абщаються со мной, хто хоче, питається наладить св’язь, общається на суржикє. (...) Рідна мова… ну якой ти общаєшся постоянно, ну імєнна твоя удобна для тебе.
    (1230^21^ – Od, lv, 21, m, h – Suržyk)
    I consider Suržyk my native language. (...) everyone communicates with me, who wants, tries to establish a connection, communicates in Suržyk. (...) Native language … well, in which you communicate constantly, well, the one that’s convenient for you.
- symbolic patriotism (PATRIO)
  1. [родной язык] український. Незавісімо от того шо я ним не разгаваріваю практіческі (...). Український язик счітаю родним, поскоку, як я сказала, я роділась в Україні, поскоку являється державною мовою і єстєствєнно являється і родною мовою моєй. (1421 – Xe, lv, 17, f, v – Suržyk)
    [my native language is] Ukrainian. Regardless of the fact that I do not speak it practically (...). I consider Ukrainian to be my native language, because, as I said, I was born in Ukraine, because it is the state language and, of course, it is also my native language.
- acquisition during childhood (ACQUI)
  1. Рідна мова – це мова, на якій ти розмовляєш з дитинства. Зараз скажу як по підручнику, але це мова, якої тебе навчила матір, а моя мама розмовляє на російській, тому моя рідна мова все ж таки російська.
    (1312 – My, st, 18, f, s – Ukrainian)
    Native language is the language you have spoken since childhood. Now I’m going to put it as in a textbook, but this is the language that your mother taught you, and my mother speaks Russian, so my native language is still Russian.
- an inclusive bundle of all components (INCL)
  1. родной [язык]... ну он больше с стороны именно, мне кажется, государства, и с другой стороны, тот, на котором тебе проще разговаривать. (...) большую часть жизни я говорю именно на нем [украинском], и все, что вокруг меня, это украинское, поэтому я считаю, что родной язык – это украинский. А русский – это просто, опять же, проще всем говорить на нем, вот почему-то так выходит. (1418 – Xe, vi, 17, f, v – Russian)
    native [language] ... well, it is more on the part of, I think, the state, and on the other hand, the one that is easier for you to speak. (...) most of my life I speak it [Ukrainian], and everything around me is Ukrainian, so I think that my native language is Ukrainian. And Russian is just, again, it’s easier for everyone to speak it, that’s why it turns out that way.

### Different conceptions of native language vs. codes as native languages

**Fig. 2:**
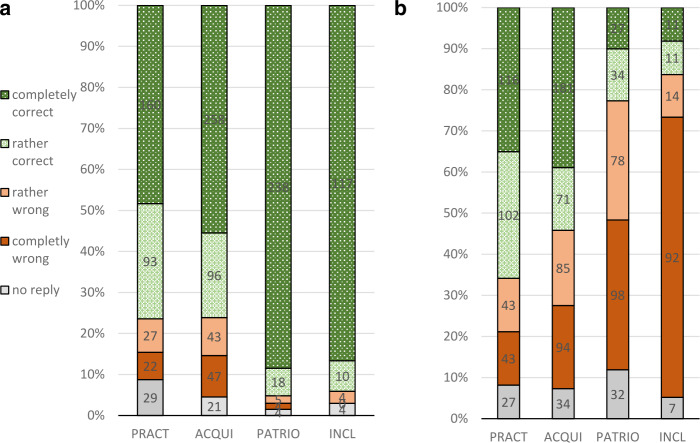
**a**: Approval or rejection of “Ukrainian is my mother tongue”. **b**: Approval or rejection of “Russian is my mother tongue”

Having determined the four groups of respondents according to their conceptions of native language, the first question to be asked is whether there are different inclinations in these four groups to declare one or the other of the three codes to be their native language. Figures 2a and 2b illustrate the major points of interest in this respect:

Here, only tables for Ukrainian and Russian are presented, because there are no significant differences between the four groups in their readiness to declare Suržyk as their native language. (We will return to the question of which aspects make respondent choose Suržyk as their native language later in the text.)

Comparing Ukrainian and Russian, the first but not most important point to be made is that Russian is much less frequently declared as the native language in all four groups. This of course relates to the fact that the number of ethnic Ukrainians among the respondents is ten times higher than that of ethnic Russians, since both groups have been considered here. Of more interest are, however, the quantitative differences between the groups for each of the two codes. Most obvious in this respect is that a patriotic (PATRIO) approach to “mother tongue” as well as an inclusive (INCL) approach almost automatically make respondents declare Ukrainian to be their native language. As has been shown in Table 2, the patriotic component is very prominent in the group INCL, too. Of special interest are of course those who unreservedly consider Ukrainian their mother tongue, and this is almost 90 percent of respondents in these two groups.

For those respondents for whom a rather realistic conception of native language is characteristic, be it oriented on current usage (PRACT) or on acquisition during childhood (AQUI), the share of unanimous acceptance of Ukrainian as their native language is clearly reduced to approximately 50 percent in both groups. When reserved approval is additionally taken into account, then three out of four respondents in the groups PRACT and ACQUI consider Ukrainian their native language. For Russian, it must first be noted that the readiness to declare it one’s native language is much lower. Apart from that, the differences between the four groups with different conceptions of native language are diametrically opposite to the constellation for Ukrainian. Only about 10 percent with a patriotic or inclusive conception of native language unreservedly declare Russian to be their native language. Within respondents for whom current usage or acquisition in childhood is most important – among them almost all of the Russians involved in the study, we observe a very clear increase in the tendency to name Russian without hesitation as one’s native language, up to 35 or 40 percent, respectively.

Briefly recapitulating this point, the first thing to note is that Russian tends to be declared the native language for symbolic or patriotic reasons on a very low level (except for respondents of Russian nationality), Ukrainian on a very high one.^22^ It should also be noted that respondents sharing the symbolic-patriotic conception of native language are in the minority, forming just one third of the respondents. For the majority of respondents, with a rather realistic conception based on current usage or acquisition, the results for Ukrainian and Russian are similar, but still with a predominance of the former.

We now turn to the question of to what extent the respondents’ estimation of their own linguistic practice, in terms of the significance of the three codes in current usage or usage during childhood, has an impact on their statement of specific “mother tongues”.

## Primary code vs. multicodalism

**Fig. 3:**
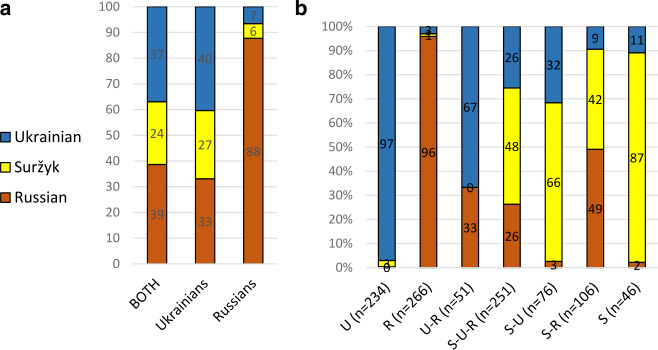
**a**: Primary codes (in %). **b**: Primary codes and speaker types (in %)

The analysis of the primarily used code by KIIS (2003), as illustrated in Map 1 above, suggests a clear dominance of Russian in the three oblasts at issue here. The average values, which additionally included data from the oblast of Zaporižžja (on the coast of the Sea of Azov), were 82 percent for Russian, 12 percent for Suržyk and only 5 percent for Ukrainian. Our results are dramatically different, cf. Fig. 3a:

Two methodological points need to be clarified before we turn to our interpretation: First, in contrast to the question about the native language, only one answer was allowed for the primary code. Multiple options were not necessary because the respondents were asked to quantify their usage of each of the three codes, which allows for a detailed comparison. And these results formed the basis for Map 2 above from Hentschel and Taranenko (2021). Second, the classification in Figs. 3a/b is three-fold. However, the partition in the questionnaire had five variants: (i) Ukrainian, (ii) Ukrainian with some Russian words, (iii) mixed language, with a rather balanced relation between Ukrainian and Russian, (iv) Russian with some Ukrainian words, (v) Russian. Then (i) and (ii) were combined into the rubric “Ukrainian” and (iv) and (v) into Russian. The intention was to make it clear to the lay respondents that rather sporadic, occasional mixing (i.e. minor insertional code-mixing in the sense of Muysken, 2000) was not at issue when asking about the mixed code.

As can be seen from Fig. 3a, the results of KIIS (2003) rather correspond to our data for respondents of Russian nationality, as more than 80 percent of them name Russian as their primary code. For respondents of Ukrainian nationality, the values for Ukrainian are even higher than for Russian, the ones for Suržyk three times higher than in the KIIS (2003) survey. As Hentschel and Taranenko (2021) argue, the differences in comparison with the KIIS survey are mainly due to the KIIS methodology. KIIS interviewed their respondents by phone, noting which code was used in their reactions. This method definitely does not measure the primary code, but the conventional code in answering phone calls (cf. Hentschel & Taranenko, 2021 for more detail). Furthermore, the much stronger position of Ukrainian in our analysis of the primary code may depend to some degree on changes in preferences for the three codes between 2003 and 2020. This will be discussed at the end of the paper.

In the following analyses it is not the primary code that is used, because it is inferior for mirroring the presence of the three codes in the tricodal society. The problem with the primary code is that for non-monocodal speakers we do not know on which grounds they select the primary code from the two or three codes they speak.^23^ The choice may even be for symbolic reasons similar to the statement of native languages. As Fig. 3b above illustrates, there is almost no methodological problem with monocodal speakers of Ukrainian or Russian. They name the corresponding code as their primary one with a share of more than 95 percent. With monocodal speakers of Suržyk (type S) this share already drops to 85 percent. One out of ten of the latter named Ukrainian as their primary code, although when asked for the frequency of using it, the estimation was less than “frequent”.

## Current usage of the codes, first language acquired and native language – comparison and interaction

**Fig. 4:**
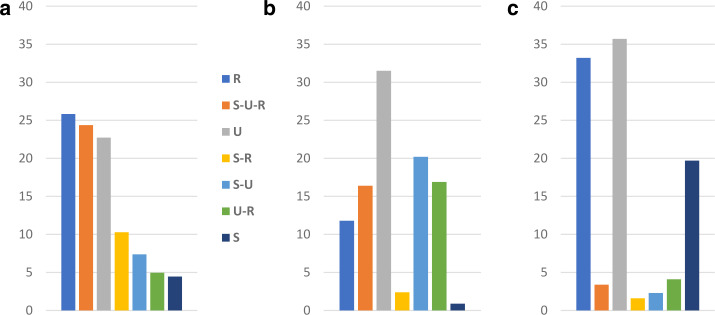
**a**: Types of speakers based on stated frequency of language use (in %). **b**: Native languages (in %). **c**: First languages (in %)

With regard to the extent of current usage of the three codes in the three Black Sea oblasts, Hentschel and Taranenko (2021, p. 284) describe that there are no “dramatic” differences. Each of the codes is in permanent or frequent use by almost one half of the respondent. Of course, there are partially very clear differences between individuals. Some are oriented on just one code, some on two codes, and others even use all three frequently. The two authors differentiate seven types of speakers (already taken advantage of for Fig. 3b above). Figure 4a illustrates the types and the share of the types (within the 1,030 respondents who provided information on the frequency of use for all three types).

These seven types correspond to the seven logically possible mono-, bi- and tricodal combinations of the three codes. As the types are based on a statistical cluster analysis of frequency estimations, a correspondence between the number of logically possible combinations and quantitatively based clusters would not necessarily be the case. A respondent from the monocodal type, say “U” for Ukrainian, usually does use the other two codes as well, but only to a quantitatively medium extent. This means that on quantitative grounds other types would be possible, e.g. “strong U and strong R” vs. “strong U and R of medium strength” etc. The names of the types are thus based on the interpretation of the results of the cluster analysis (cf. Hentschel & Taranenko, 2021, pp. 285–287 for details).

It is obvious that the presence of the seven speaker types in the Black Sea region shows a bipartite constellation (Fig. 4a). Three of the seven types have values of about 25 percent. These are respondents with monocodal preferences for Russian or Ukrainian in everyday life and respondents that name all three codes as frequently used ones. The four other types show shares from roughly 10 percent down to roughly 5 percent. These are all three speaker types with a strong bicodal practice and those who practise almost exclusively Suržyk. The low share of monocodal speakers of Suržyk compared with the monocodal Russian and Ukrainian type is not astonishing, because Suržyk is principally a vernacular used in diglossic (triglossic^24^) constellations with Ukrainian and Russian in unofficial, private communicative settings. In more public settings the acceptance of Suržyk is rather low (cf. Hentschel & Zeller, 2016; Bilaniuk, 2018 and Sect. 5 below).

There are some specific peculiarities of the speaker types observed for respondents with self-ascribed Russian nationality: First, there are almost none (two out of 122) among the monocodal speakers of Ukrainian. Second, two thirds of them are monocodal speakers of Russian, but, third, these respondents make up just one quarter of all monocodal speakers of Russian.

For the seven types of speakers (respondents), based on their estimations of linguistic practice in everyday life, now the statements on their native languages will be compared and, in a second step, their statements about the first language or code they grew up with. Figures 4b and 4c respectively illustrate the results.^25^ Already the shape of the three figures shows that there is a large degree of mismatch between the respondents’ perspectives on the three codes as frequently used codes, as native languages, or as the code of socialization during childhood.

### Frequently used code vs. native language

We start with the three largest of the seven groups of speaker type, which make up (with rather equal shares) almost three quarters of all of the 890 respondents who provided self-quantifications on the use and the status of native language for all three codes:

i. Speaker type U (218 respondents): The vast majority, eight out of ten of speakers of the speaker type U (almost only Ukrainian), names only Ukrainian as their native language, one in ten names Ukrainian together with Suržyk. As has been mentioned above, there are almost no respondents with Russian “nationality” in this speaker type,
ii. R (210): Monocodal users of Russian reveal a heterogeneous picture of “mothertongueness”. Only four out of ten report Russian to be their only native language. For the ethnic Russians among them, the relation is however eight out of ten. Four in ten from both ethnic groups in speaker type R name Russian together with Ukrainian, one in ten name only Ukrainian. This all implies that monocodal speakers of Russian with Ukrainian nationality (two thirds of monocodal speakers of Russian) preferably declare Ukrainian as their native language, although they rarely (if at all) practise it. Only a quarter of them unreservedly names only Russian as their native language, almost half of them Russian and Ukrainian, one in six only Ukrainian. With the latter group there is thus a complete mismatch between native language and current usage of codes.^26^
iii. S-U-R (216): Of the speakers with a declared “tricodalism”, indeed more than one third name all three codes as their mother tongue. The share of those naming Ukrainian and Suržyk (but not Russian) as their native languages is a little smaller. The remainder name Ukrainian and Russian or only Ukrainian with comparable frequency.

With regard to other speaker types, a quantification is less meaningful due to small numbers. It suffices to say that speakers of the S-R type (96) mostly name all three codes as their native language, (similarly to S-U-R speakers), to a lesser degree Suržyk and Ukrainian (but not the combination of Suržyk and Russian). S-U speakers indeed preferably name Suržyk and Ukrainian or Ukrainian alone.

From the point of view of respondents’ statements about their mother tongue one can make the following statements about native language:

i. Native language only Ukrainian (280 respondents): Six in ten declaring Ukrainian to be their sole native language belong to the monocodal Ukrainian speaker type (U). The other speaker types are represented to a much less extent: by either one in ten or by even fewer.
ii. Only Russian (105): Almost nine out of ten are indeed monocodal speakers of Russian (R), one in ten uses Russian and Suržyk frequently (S-R).
iii. Ukrainian and Russian (146): One half belongs to the monocodal Russian speaker type (R), about two in ten are each either bilingual speakers of Ukrainian and Russian or tricodal speakers.
iv. Ukrainian and Suržyk (180): This group is very heterogenous in terms of speaker types: Four out of ten are tricodal speakers, two out of ten come from the S-U type, one from each of the monocodal types U, R, S.
v. Ukrainian, Russian and Suržyk (150): One half consists indeed of tricodal speakers, a good quarter of S-R speakers and one out of ten is a monocodal S speaker.

The number of speakers of other combinations are too small to be commented upon.

The general interpretation of these observations is as follows: Among those people in whose daily linguistic practice Russian, Suržyk or both codes play a major role, there is a considerable reluctance to name Russian as their native language (apart from those with Russian nationality), an even stronger one to name Suržyk (cf. Hentschel & Zeller, 2016 and Zeller, 2022 in this volume). On the basis of the different layman’s approaches to the native language this should already have been expected for Russian, for Suržyk this becomes clear only here. If Russian or Suržyk are named as native languages by respondents with Ukrainian nationality, this occurs preferably in combination with Ukrainian.

### Frequently used code vs. first language (code)

i. The most obvious phenomenon is that combinations of codes play only a peripheral role in first language (code) acquisition (cf. Fig. 4c). This is far from unexpected: First, it is a common phenomenon that there is only one “family code”, at least between parents and children. Regarding combinations of codes, that of Ukrainian and Russian is most frequently named here, however, with a low share of 4 percent. It is relevant in this context that the share of respondents with one parent being ethnic Ukrainian and the other Russian is rather low, roughly 10 percent.
ii. Ukrainian and Russian are named as the only first language to a more or less equal extent of roughly one third of the respondents. Suržyk is named by one fifth. It is presumably safe to assume that at least some of the respondents were reluctant to admit that they grew up with only Suržyk in their family. This vernacular is stigmatized – as has been mentioned above – as the code of the uneducated, especially when there is no “literary” variety used as well. One might feel uneasy to reveal that this was the case in one’s family. This means that all three codes might be on a similar level as the first linguistic code acquired, although Suržyk is clearly named less frequently than Ukrainian and Russian.

Regarding the ethnic groups of the respondents, we observe that among Russians more than two thirds declare only Russian as their first language (the rest is distributed rather evenly over other constellations of first languages), among Ukrainians more than a quarter do so. This means, on the other hand, that among those who in the first years of their lives were socialized only in Russian there are four Ukrainians and only one Russian.

### The presence of the codes in the respondents’ surroundings

**Fig. 5:**
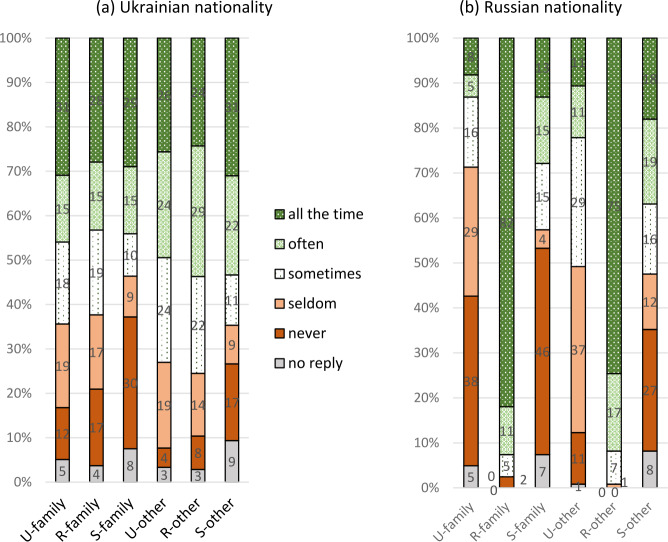
The presence of the three codes in the family and in other everyday contexts of the respondents

This brings us back to the question of the extent to which the three codes are currently used “in reality”. Up to this point we have only discussed data based on respondents’ self-estimations of their own linguistic practice. To a certain, but of course variable degree people are interested in presenting themselves in a good light, which is especially to be reckoned with for the symbolic aspects of the “mother tongue”, which for many, as has been shown, has a symbolic-patriotic significance. A similar approach cannot be completely ruled out even for statements on the codes frequently practised or on the ones acquired during childhood. We therefore now turn to the question of how respondents estimate the presence of the three codes in their surroundings. First, the situation in families will be looked at, which is of course a closer or more intimate social context for the respondents and, second, the situation outside the family. The corresponding questions were “How often is X used or to be heard?” The presentation of the quantitative relations will be differentiated for respondents with Ukrainian and with Russian nationality, because they differ clearly between the two groups. Figure 5 presents the results:

The first thing to notice in this analysis is that there are very few refusals to answer this question. This underlines that personal statements in surveys can be influenced by a feeling of discomfort for some phenomena. The second point to be made is of course the differences between ethnic Ukrainians and ethnic Russians. It looks as if the two groups are living in two separate linguistic worlds. Nearly one half of the respondents of Ukrainian nationality (Fig. 5a) report the frequent or very frequent usage of Ukrainian, most interestingly a little more often for non-family (“other”) contexts. Apart from that, first, the same holds for Suržyk, and second, the results for family contexts and non-family contexts do not differ greatly. An interesting point is connected with the presence, or to be precise, the absence of Suržyk in the micro-world of this group: 30 percent state that it is not used in their families, but only 17 percent say that they are not confronted with it in other contexts. With respondents of Russian nationality (Fig. 5b) this discrepancy is even clearer: 46 vs. 27 percent. For both groups it is obviously “easier” to acknowledge the presence of Suržyk outside their families, i.e. at some distance from themselves. This can be interpreted in the sense that the usage of Suržyk is to a certain degree even more widespread than people are ready to admit regularly using it themselves.

The most striking difference between the two ethnic groups is of course connected with the presence of Russian in their respective micro-worlds. More than 90 percent of the ethnic Russians state that it is frequent, mostly very frequent in their surroundings, both in their families and in other social contexts. In view of the widespread monocodalism of ethnic Russians already described above, this is of course less astonishing for family contexts than for other ones. The corresponding figures for ethnic Ukrainians are much lower: a little more than 50 percent use Russian frequently or very frequently.

This leads us to the question of competence in Ukrainian and Russian and to the question of shifts from one language or code during the respondents’ lifetimes.

## Competence in Ukrainian and Russian

Since there are relatively large groups that neither use Ukrainian nor Russian frequently, the question of competence in the two languages arises.^27^ Eight in ten respondents state that they can speak and write Ukrainian fluently. Almost everybody reports that they can freely understand spoken Ukrainian and nine out of ten claim to be able to read long texts in Ukrainian. With regard to Russian, these figures are even slightly better. Thus, the differences between Ukrainian and Russian are rather subtle, with a slight quantitative advantage for Russian.

**Fig. 6:**
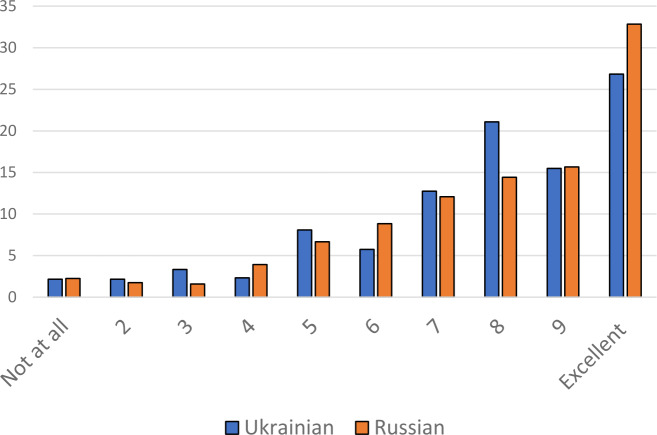
Self-judgements on competence in Ukrainian and Russian (in % for 1,200 respondents)

The judgements about the level of competence in the two languages without distinguishing between active and passive competence in oral or written form are more informative, as the next figures illustrate:

As a matter of fact, even these quantitative data in the general overview of Fig. 6 evidence a balanced relation between Ukrainian and Russian: For both languages the share of respondents judging their competence with a score of 7 or worse is 37 percent. However, among those who rate their competence better than 7, an excellent command of Russian is stated more often than an excellent one of Ukrainian. On the other hand, a score of 8, i.e. the grade “good”, is more frequent for Ukrainian, with the grade “very good” for both on the same level.

**Fig. 7:**
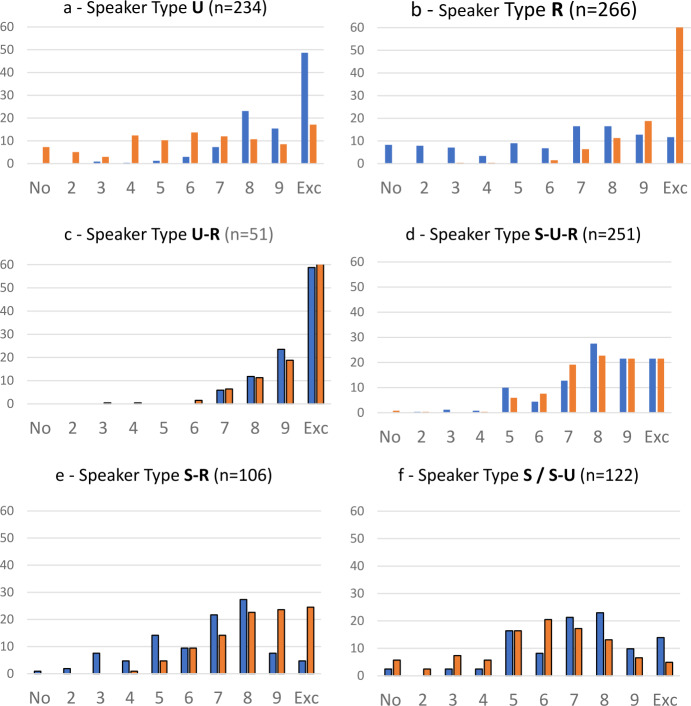
**a** to **f**: Speaker type and competence in Ukrainian and Russian (colours as above: blue – Ukrainian, orange – Russian) (Color figure online)

Clear differences in the self-judgements on competence in Ukrainian and Russian become obvious when speaker types are discriminated; cf. Fig. 7:

It is obvious that many monocodal speakers of Ukrainian report a rather reduced competence in Russian (Fig. 7a) and vice versa for the Ukrainian of monocodal Russian speakers (b). And these two groups make up roughly one half of all respondents. Respondents who named Russian and Suržyk as frequently used codes report similar levels of competence to monocodal speakers of Russian (e). Here as well, competence in Russian is obviously better than that in Ukrainian, but of course not as clearly as in the case of monocodal speakers of Russian. A general point that can be made for all speaker types with Suržyk as one of the frequently used codes (from d to f) is that the self-estimated level of competence in Ukrainian and Russian is much lower than for monocodal speakers or for the small group of self-stated balanced Ukrainian-Russian bilinguals (c). A balanced Ukrainian-Russian competence level can be observed with one other group, namely with those who use all three codes, including Suržyk, with relatively high frequency (d). And this is as large a group as the two monocodal groups. Last but not least, there are speakers with only Suržyk or Suržyk and Ukrainian in frequent use, who by and large tend to report a slightly higher competence in Ukrainian (f), which is slightly more obvious with those who report Ukrainian as well. However, in both subgroups the picture is rather diffuse.

So far in this section, the two ethnic groups have been analysed together. What has to be added is a word on competence in Ukrainian among respondents of Russian nationality, almost all of whom, as has been outlined above, belong to the monocodal Russian speaker type. For this reason, a differentiation based on speaker types would not be meaningful. It is, however, possible to make a more precise comment on the results presented in Fig. 7b: For ethnic Russians and Ukrainians in this group together, competence in Ukrainian is not high, and it is not surprising that it is worse for speakers of Russian nationality than for those of Ukrainian nationality. More than half of the ethnic Russians in this group has a poor level of competence in Ukrainian with a score of 5 or less on the 10-point scale (a quarter even has no competence at all). On the other hand, only 14 percent of ethnic Russians claim to have a very good or excellent competence (score 9 and 10) and 28 percent of ethnic Ukrainians.

These data hint at a situation in which Suržyk is to a certain degree some sort of communicative bridge in everyday life between speakers oriented towards Ukrainian or Russian, at least for ethnic Ukrainians. This is mirrored in the answers to the aspect of tolerance towards the three codes. There were two questions in our survey on this topic: “Have you ever felt embarrassed because of using X?” and “Have you ever been jeered because of using X?” Both questions were asked for all three codes. There are extremely few positive answers on either question. Three out of 100 respondents have experienced occasions of embarrassment for using Suržyk, one of 100 for using Ukrainian and even fewer for using Russian. Still fewer experienced objections to their choice of codes, interestingly more for Ukrainian (15 out of 1,000) than for Russian or Suržyk (7 and 8 of 1,000).

In interviews, respondents were more likely to mention good-natured ridicule than real bullying:

(5) це [суржик] деколи викликає сміх, aле такий позитивний, ти з цього посміявся і тобі ну..., не так, що “га-га-га” насміхаєшся. А це просто прикольно, навіть деякі слова можеш перейняти і потім так само їх використовувати.
  (1237 – Od, lv, 21, m, h – Ukrainian)
  It [Suržyk] is sometimes laughable, but rather positive, you laughed at it and you, well ..., not that you would go laughing “ha-ha-ha”. And it’s just cool, you can even adopt some words and then use them in the same way.

In most cases, according to the respondents, it is Suržyk that is stigmatized, but in some cases the use of the Ukrainian (6) or Russian (7) languages also causes ridicule or objection.

(6) У нас получається в мене в сестри чоловік, він – руский, сам з Кемеровской області, но він поначалу нас не понімав трошки, а потом він уже привик, хоч він сам руский, і вже йому і за сємдесять, і як він не хотів перевести, як він не хотів промовить ці слова українські, то в нього всьо равно ніяк. То ми з нього глузували, а не він з нас. (1432 – Xe, st, 65, f, v – Suržyk)
  We have, therefore, my sister’s husband, he is Russian, himself from the Kemerovo region, but at first, he did not always understand us, and then he got used to it, although he is Russian, and he is over seventy, and as he wanted to translate, as he wanted to say these words in Ukrainian, he anyhow did not succeed. Then we made fun of him, not he of us.

(7) Я работала в сфєрі обслуживанія, і я начала балакати, як я балакаю, то дєвочка одна мені сказала так: “нє разгаварівайтє са мной на украінскай мовє, разгаварівайтє са мной на русском язику”. (1437 – Xe, lv, 35, f, v – Suržyk)
  I worked in the service sector, and I started talking as I talk, and one girl told me: “do not talk to me in Ukrainian, talk to me in Russian.”

Respondents often even show self-confidence, despite ridicule of their language.

(8) В унівєрсітєті, то да, ми приїхали з села, то в нас такий тута мєсний діалєкт, можна сказать. Уже Кривий Рог, город, всі по-русски там, то з нас, канєшно, всі підсміювалися, і ми як починали по-українськи, нам трошки було неудобно, і ми начинали, ето ж перві курси, під них підстраюваться, ну, балакали по-російськи. Ну а потім уже як трошки стали старші, то ми рішили, чо ми должні під них підстраюваться, нехай лучше вони під нас. І ми начали розмовлять по-своєму і, ну, і перестали, коли вони з нас сміялись, ми просто на це відповідали: хлопці, ви лучше підстраюйтесь під нас, українська Україна, українська мова, ну, тобто в таком плані. (1443 – Xe, st, 42, m, h – Suržyk)
  At the university, yes, we came from the village, we have such a local dialect here, as one can say. Kryvyi Rih, on the other hand, the city, all in Russian there, then of course, all of us laughed, and we started in Ukrainian, we felt a little uncomfortable, and we started – those were the first years of studying – to adapt to them, well, chatted in Russian. Well, then as we got a little older, we decided that we shouldn’t adapt to them, but rather they to us. And we started talking in our own way and, well, we stopped when they laughed at us, we just answered: guys, you better adapt to us, Ukrainian Ukraine, Ukrainian language, well, that is, something like that.

However, the number of instances of ridicule or objection described above is far too small to interpret their differences. What can be interpreted is the low level of embarrassment and objections regarding the practice of the codes, even for Suržyk. Of course, there are conversational situations in which one of the codes is more appropriate or inappropriate. And discomfort may result if expectations for the use or non-use of a code are not fulfilled. This seems however to be bound to specific situations rather than general attitudes in society (see Zeller, 2022 in this volume on attitudes).

## Language or code shifts

It is a well-known fact that language policy in the independent Ukrainian state has enforced the position of Ukrainian in society,^28^ especially in official or public contexts. Ukrainian is the only state language, but Russian has a legally fixed status on the regional level.^29^ As Hentschel and Brüggemann (2015) have outlined for Central Ukraine^30^ in the year 2014, (i) this situation is obviously supported by a majority of the population, (ii) there is neither an aversion towards Russian in society, even with people who are clearly oriented towards Ukrainian, nor a prosecution of speakers of Russian, (iii) people do not see a language conflict in society, the emotional discussion among political and cultural elites notwithstanding, and (iv) that people prefer to have the specific official status of Russian and other languages fixed rather on the regional level of oblasts. This holds even for the oblast of Xarkiv, with its clear orientation towards Russian in 2014, though to a somewhat lower degree.^31^ Of course, due to the cruelty of Russian warfare, these attitudes will probably change to the negative for Russian, at least to a certain extent.

Ukrainian, without any doubt already before 24 February 2022, had become more important for many people in order to live a normal professional and social life, most probably with differences between regions such as, for example, between Lviv, as strongly oriented towards Ukrainian on the one hand, and Xarkiv, as strongly oriented towards Russian, on the other hand. Nevertheless, the question arises of the extent to which people shifted to Ukrainian, especially in regions where during Soviet times Russian clearly predominated, as was clearly the case in the three oblasts on the Black Sea coast.

**Fig. 8:**
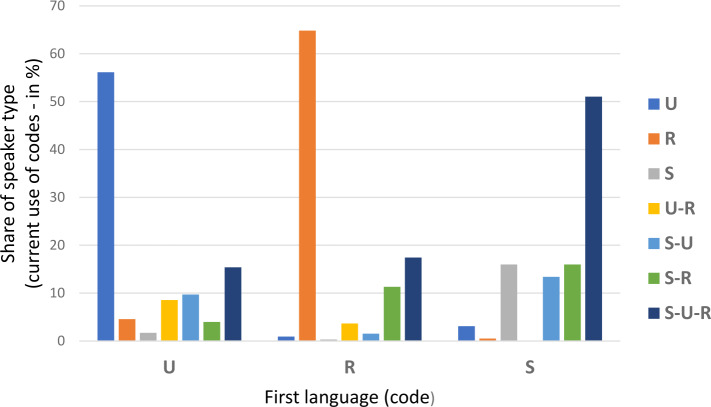
Shifting codes: From first language (code) to speaker type

We will try to shed light on this question by analysing how the respondents’ linguistic practice developed after their, as a rule, obviously monocodal first linguistic socialization (cf. Fig. 4c). To this end, we will analyse which type of speaker (cf. Fig. 4a) they now report to be. The number of respondents reporting multicodal linguistic socialization during childhood is too low in the four groups to allow for a meaningful quantitative analysis. Figure 8 illustrates the correspondences for the three monocodal groups:

The major observations are as follows:

- The majority of respondents reporting a monocodal first language Ukrainian or Russian state that they are monocodal in their current use as well – to a similar extent for both languages: almost 60 percent for Ukrainian und somewhat more than 60 percent for Russian.
- This is different for respondents with monocodal Suržyk: Only roughly 15 percent stay with Suržyk alone in their daily linguistic practice. This, of course, was to be expected, because a vast spectrum of conversational settings apart from family, friends, and maybe neighbours demand the practice of one of the acknowledged literary (in the sense of standard) languages.

Here again, several utterances from the in-depth interviews are offered as illustration:^32^

(9) Когда я была маленькая, в нашей семье все общались на суржике – мама, папа, бабушка, дедушка. Тому і я, в детстве і зараз, разговариваю на суржику. (…) я і зараз общаюсь на суржику, і з вами. (…) я разговариваю так як мені удобно, якщо вже мене не понімают, то я намагаюсь перейти на той язик, на який треба – русский чи український. Хоть мені це іногда буває сложно.
  (1310 – My, lv, 33, f, v – Russian / Suržyk).
  When I was a kid, everyone in our family spoke Suržyk – mom, dad, grandma, grandpa. That’s why I, as a child and today, I speak Suržyk. (...) I still communicate in Suržyk, even with you. (…) I speak as I feel comfortable, if somebody doesn’t understand me, I try to switch to the language I need – Russian or Ukrainian. Although it is sometimes difficult for me.

(c) The above quotation provides an explanation for the enormous shift among speakers with exclusively Suržyk as the first code towards all three codes in everyday linguistic practice (S-U-R). This is simply a social necessity for communicating with the large number of speakers who practise only one of the two “real” languages.
  1. Мій основний язик – це змішана, ну ілі суржик. З дєтства мене учили разговарювать зразу синхроно на двох язиках. Я считаю, що це прекрасно, потому шо щас це мені дуже сильно помагає, я можу переходити з руского на українский, з українского на руский (...) Мені вообще це комфортно.
    (1240 – Od, vi, 20, f, h – Suržyk)
    My main language is mixed, well, or Suržyk. Since childhood, I was taught to speak synchronously in two languages. I think it’s great, because right now it helps me a lot, I can switch from Russian to Ukrainian, from Ukrainian to Russian (...) for me in general it’s comfortable.
(d) Speakers with exclusively Suržyk as their first code almost never give up practising Suržyk frequently. The number of respondents reporting only one code, Ukrainian or Russian, in their everyday lives tends to zero, the number of respondents using Ukrainian and Russian (but not Suržyk) is zero. Quite a few of them report one of the combinations of Suržyk with Ukrainian or Russian (S-U, S-R) as their major codes: roughly 15 percent each.
  1. Ну, я з самого дєтства балакаю на суржику, мені зараз восємнадцять год, і тута шоб сказать, шо на якихось опредєльонних – нє, у нас іногда проскакує і українська мова, і російська, і то все якось, якось вперемєшку.
    (1440 – Xe, lv, 18, f, s – Suržyk)
    Well, I’ve talked in Suržyk since very early childhood, I’m eighteen years old now, and here one can say that in certain [languages] – no, sometimes people hop, between Ukrainian and Russian, and all this is somehow, somehow mixed up.
(e) The S-U-R-type of frequent current usage makes up the second largest group in the case of monocodal linguistic socialization with Ukrainian or Russian (15 or 18 percent), after the majority groups of monocodal-oriented speakers of Ukrainian and Russian. For both groups of the previously monocodal Ukrainian- or Russian-speaking children, according to (e) and (d) Suržyk becomes a frequently used code in further stages of their lives. It is well known that even people with a linguistic socialization in one of the literary languages during childhood and an academic education under certain conditions have to turn to a (mixed) vernacular, for example in professions such as engineers, architects or veterinarians to be accepted by workmen or farmers.
  1. Підприємці у нас всі суржики. Ну як частина єсть і російськомовні, єсть і україномовні. Ось, до речі, оці люди, вони україномовні. Але я вам скажу, а на суржик перейшов, ось підприємець, хлопчина оцей. Він україномовний, він з Тернополя, але на суржик перейшов.
    (1427 – Xe, lv, 53, f, h – Ukrainian)
    Entrepreneurs are all “Suržyk-folk”. Well, partially there are Russian-speaking ones, there are Ukrainian-speaking ones, too. By the way, these people are Ukrainian-speaking. But I’ll tell you, there’s one who has shifted to Suržyk, an entrepreneur, this guy. He is Ukrainian-speaking, he’s from Ternopil, but he shifted to Suržyk.
(f) Apart from the group of current S-U-R speakers, a switch from Russian to Ukrainian is almost absent for speakers with only Russian in their first linguistic socialization – compare the very low share of U-R speakers. By the way, this is a clear indication of the fact that there is no pressure on the Russian-speaking population of Ukraine to abandon their code of first choice. It is even the case that there is a slight tendency for monocodal Ukrainian children to later turn to Russian as well. However, this might be a reflexion of the former predominance of Russian in public life.
  1. Я думаю, що ті, які приїжджають вони не сильно можуть пристроїтись, бо вони виросли на тому язику. А єслі людина виросла на чисто українськом язику чи на чисто руском, то їй тоже тяжко перейти на суржик. (…) Я знаю таких людей, які тут живуть годами і вони не перейшли на суржик полностю. Так на руском вони й говорять, не можуть перейти.
    (1224 – Od, lv, 47, f, h – Suržyk)
    I think that those who come here, they cannot adapt that much, because they grew up in that language. And if people grew up in pure Ukrainian or in pure Russian, it is also difficult for them to switch to Suržyk. (…) I know people who have lived here for years and they have not completely shifted to Suržyk. They still speak Russian, they are not able to switch.
(g) Last but not least, it should be realized that people with a monocodal Ukrainian background have more readily turned to only Russian in current usage than vice versa. On the other hand, those with a monocodal Russian background more readily tend to add Ukrainian to their repertoire of frequently used codes than those with monocodal Ukrainian background add Russian to it.

Thus, generalizing these observations, we can state the following: If there has been a shift among people to Ukrainian, which is more than probable but cannot be determined here precisely, then people with Russian or Suržyk as their code of first linguistic socialization add Ukrainian to their repertoire, rather than abandoning their first code completely. A further detail to be added in connection with this generalization is that there are almost no differences between respondents of Ukrainian or Russian nationality in this respect: If their first language was Russian, which was almost exclusively the case with ethnic Russians, they maximally add Ukrainian or (more often) Ukrainian and Suržyk to their repertoire, more or less to the same extent. Whatever shifts have been accomplished by people during their lifetimes, these were most obviously not caused by social pressure from people with a different linguistic orientation.

## Summary, conclusions, questions for future research

The region this study concentrated on belongs to the primary territorial aims of the war being waged by Russia against Ukraine. In the article, we tried to present basic insights on the linguistic situation in 2020 / 2021 in the three oblasts of Odesa, Mykolaïv and Xerson, which are traditionally seen as a region of Russian language predominance, even by Ukrainian colleagues from social sciences. As has already been pointed out by Hentschel and Taranenko (2021), this view must be adjusted to a large degree. As a matter of fact, Russian still is in a strong position. Almost one fifth of ethnic Ukrainians (and nearly all ethnic Russians) belong to the group of monocodal speakers of Russian. However, the quantitative dominance is much less clear than previous analyses like that by KIIS (2003, cf. Map 1) suggest. The abovementioned more recent analyses by Ukrainian researchers from the social sciences point in the same direction (cf. Razumkov Centre, 2016; KIIS, 2019), but due to methodological shortcomings, pointed out by Hentschel and Taranenko (2021, pp. 273–275) do not offer a precise picture, not least due to the neglect of Suržyk.

The sections above contain a wealth of quantitative data and the main points will be summarized and interpreted here comprehensively.

The balanced tricodalism that Hentschel and Taranenko (2021) have emphasized is even more visible when the usage of codes in the respondents’ surroundings is asked for instead of their own linguistic practice. The two authors based their assessment on self-estimations of the extent of usage of the three codes. For all three codes the respondents report that in their families and in their micro-world outside their families they are practised often or even all the time with shares near to 50 percent or even a little higher. The quantitative relations between the three codes are more or less balanced. The only clear exception from balanced quantitative relations between the three codes in the everyday life of people on the Black Sea coast is that far more respondents report never using Suržyk in their surroundings than Ukrainian and Russian. This is plausible to a certain extent if we assume that a considerable number of the respondents live in contexts where “only” one or both of the literary languages are practised, most probably in socially “high” professional surroundings. On the other hand, that Suržyk is reported as never being used in family contexts and in other social surroundings prompts some doubts, as Suržyk is generally seen as a variety mainly practised in the family or with rather intimate acquaintances. The social conditions of the described relations still need to be analysed.

It is clear that with speakers of Russian nationality, who made up one tenth of all our respondents, there can be no question of a balanced tricodalism. Their language is predominantly Russian: As their native language, as their most frequently used language and even as the language being used and heard in their daily surroundings – in each respect with a more or less clear tendency to exclusiveness. This group of respondents seems to live to a large degree in a linguistically different social world, as do some of the monocodal Russian-speaking Ukrainians.

In regard to the question of shifting from one code to two or three codes, this study mirrors rather precisely that there is a certain shift to Ukrainian in current linguistic practice. For the majority of those with a monocodal Russian background during childhood, Russian definitely remains the only frequently used language. This is most extreme for respondents of Russian nationality: only a few have added Ukrainian or Ukrainian and Suržyk to their linguistic repertoire. Among the ethnic Ukrainians with a monocodal Russian background during childhood, almost one quarter reports a frequent use of Ukrainian as well, often accompanied with a frequent practice of Suržyk. The self-estimated competence in Ukrainian in this group is considerable. It can (for the moment) not be discerned to what extent this reflects a shift to Ukrainian, as it may be the case that younger respondents could take advantage of better teaching of Ukrainian in the education system. Nevertheless, it is more than obvious that hardly anyone with Russian as the only code used during childhood abandoned it, not even ethnic Ukrainians.

Apart from the “competition” between Ukrainian and Russian in Ukraine society, it can be stated that Suržyk holds onto its position in the “architecture” of linguistic codes pretty well, even on the Black Sea coast. Our figures suggest that it was (at least) on a comparable level with Ukrainian and Russian as the code of linguistic socialization during childhood. For approximately one half of our respondents it belonged to the frequently used codes even in later years, though only rarely as the only one in frequent use. Last but not least, people estimate the presence of Suržyk as much stronger outside their families.

The most significant shift to be observed was that in the statement of their native language by ethnic Ukrainians. In the Soviet census of 1989 and in the only Ukrainian census of 2001, ethnic Ukrainians in the three oblasts at issue here clearly named Ukrainian less frequently as their native language than in our survey. In the former census, only little more than one half of the population did so, here it was up to nine out of ten speakers. The language policy in independent Ukraine has obviously at least tightened the symbolic-patriotic link between titular ethnic group and the “titular” language. That linguistic practice changes much less rapidly, is, of course, not astonishing.

This study, of course, prompts many further questions. Almost all results presented here must be related, for example to sociodemographic criteria, such as age, sex, education, profession, diverse political and linguistic attitudes etc. This will be undertaken in the project underpinning this paper in the near future by employing multifactorial statistical methods to a much larger extent and by deeper qualitative analyses of the material gathered. Only then will further interpretations of the empirical results be possible. Nevertheless, we hope to have given a rather differentiated description of the linguistic landscape.

However, none of the figures reported above, especially the reports about the general lack of objections and ridicule of their usage of any code (apart from rare exceptions), support the Kremlin assertion of prosecution of parts of the population for linguistic reasons. Almost no one claimed that their usage of Russian was objected to by others; not more or even fewer claimed to have experienced objections to their speaking Ukrainian or Suržyk. Of course, there are different opinions about the enforcement of Ukrainian by the state government and the legal status of Russian in the Ukraine (cf. Zeller, 2022, this volume). However, after at least one and a half centuries of severe neglect if not suppression of Ukrainian under Russian rule, be it Soviet or Tsarist (cf. again Danylenko & Naienko, 2019), the political enforcement or “positive discrimination” (as one may call it in the words of western gender policy) of Ukrainian in the state as a whole should be acknowledged as a more than understandable and legitimate linguistic policy.

The linguistic situation in the three Black Sea oblasts in independent Ukraine can by no means serve as a legitimation for the Kremlin war in the sense of saving Russians or Russian-speaking people from prosecution. The assertion of such prosecution, if not worse, is one of the masses of lies that the Kremlin regime has spread over the last decades. Unfortunately, it is obviously not only a vast majority of the Russian people that is ready to believe these lies, but even a large group of “Putin-versteher” in the western world that has shown such inclinations, at least until recently.

The ongoing war will change many things, including linguistic practice and attitudes as well. The situation which we have just described for 2020 / 2021 will most probably change radically.

## Footnotes

1 An excellent account of the history of Ukraine, also in its relation to Russia and Poland, can be found in Kappeler (2019). Cf. especially Chaps. 4 to 7 regarding the region at issue in this paper.

2 On the role of “Novorossiya” in Russian nationalism cf. Laruelle (2019, pp. 195–213).

3 Cf. the institution’s website: https://www.kiis.com.ua/?lang=eng and the bibliography at the end of the paper for the specific analysis. There are more recent analyses by Ukrainian researchers from the social sciences (cf. for example KIIS, 2019 or Razumkov Centre, 2016), which illustrate a somewhat higher share for Ukrainian in the region discussed here. Their strengths and weaknesses are discussed by Hentschel and Taranenko (2021, pp. 273–275).

4 In view of the stigmatization of the terms “Suržyk” and “Trasjanka”, not least by Ukrainian and Belarusian linguists (cf. Hentschel, 2017 and below), the same author Hentschel (2013) proposed in one of his early works on Belarusian Trasjanka to avoid using them and instead to use neutral formulations such as “Belarusian-Russian mixed speech”, abbreviated as BRMS, and analogously URMS for Suržyk, which he used in his studies. The reactions of the community working in this field were twofold. Some appreciated this terminological proposition, others rejected it as being clumsy. Furthermore, they maintained that the old terms were well known and may be employed in research papers in a neutral way. Ultimately, Hentschel and his Oldenburg group decided that the latter group of colleagues were right and returned to the traditional terms, using them neutrally, without any connotation, as will be the case here. Moreover, as was hinted at by Bilaniuk (2018), the negative attitudes towards mixed speech in Ukraine are retreating to a certain degree in Ukrainian society, though obviously not among some linguists and representatives of the cultural elites in Ukraine. It should also be underlined that traditionally “typical” Suržyk is seen as a so-called Ukrainian-based mixed speech (cf. Flier, 2008). It is characterized by the social fact that its speakers or the ancestors of its speakers used to speak autochthonous Ukrainian dialects before moving into towns and cities where more Russian was spoken. Its grammatical traits (e.g. inflectional patterns) are predominantly Ukrainian (cf. Menzel & Hentschel, 2017 and Hentschel, 2018a, 2018b). In this study we use “Suržyk” in a somewhat broader sense, including mixed speech with a stronger impact from Russian, too. This corresponds with the layman’s understanding of the mixed code. The differentiation of Ukrainian-based “typical Suržyk” from Russian-based “Neo-Suržyk” is one of the aims of the first research project mentioned in Footnote 9 below (cf. Hentschel and Reuther (2020 for more details).

5 For further information cf. Hentschel and Taranenko (2015, 2021), Hentschel (2018a, 2018b) or the anthology edited by Hentschel et al. (2014). The latter volume offers a comprehensive overview of the literature on Suržyk (and Trasjanka).

6 Cf. Danylenko and Naienko (2019) for the history of the suppression of the Ukrainian language under Russian rule.

7 A similar, but not as elaborated approach, had been already presented in Hentschel and Taranenko (2015).

8 This work is mainly based on two grants: (1) *Hybridization from two sides: Ukrainian-Russian and Russian-Ukrainian code-mixing in the context of the sociolinguistic situation in Southern Ukraine along the Black Sea coast*, funded in an Austrian-German cooperation by the *FWF – Der Wissenschaftsfonds* (no. I 4189-G30) and the *Deutsche Forschungsgemeinschaft* (DFG, no. 419468937), headed by Tilmann Reuther and Gerd Hentschel respectively. This still ongoing project concentrates on the Ukrainian Black Sea coast. For some comparisons with the situation in Central Ukraine, data from a completed project headed by Gerd Hentschel have been considered: (2) *Variability and stability in a mixed substandard in extensive and time-stable language contact: The Ukrainian-Russian Suržyk in Ukraine*, funded by the Fritz-Thyssen-Foundation (no. 10.14.1.066).

9 Unfortunately, the differences will not be mirrored in the black-and-white printed version of *Russian Linguistics*. We politely ask the reader to consult either the online version of this issue or the following URL: https://uol.de/f/3/inst/slavistik/projekte/Schwarzmeer/MapStrenghtofCodesURS.PNG.

10 Unfortunately, the oblasts of Luhans’k, Donec’k and Zaporižžja could not be considered in the 2019/2020 survey due to the war-like situation, especially in the first two, after 2014.

11 In stating this it must be underlined that metropolises with more than a million inhabitants were not considered in either research project mentioned above. This is due to the specific orientation of the abovementioned projects on Suržyk. Metropolises in eastern and southern parts of Ukraine are traditionally seen as Russian speaking, with a rather insignificant share of Suržyk. Thus, if the city of Odesa, the only metropolis in the Black Sea region, would have been included as well, the share of Russian in the oblast of Odesa would certainly have been higher. In the project on Central Ukraine the metropolises Kyïv, Xarkiv and Dnipro were not considered. If, however, they would have been included, the colouring of these oblasts in Map 2 would have slipped slightly toward red.

12 It should be noted that the absence of data on the metropolis Odesa makes the figures more directly comparable to those from the other two southern oblasts because the latter do not have such a large urban centre. The same would hold, of course, for Central Ukraine, where there are metropolises of more than 1,000,000 inhabitants in only three oblasts.

13 Since the index for the use of the three codes was standardized to a maximum of 100, arithmetically these figures may be directly compared with percentage values with the same maximum of 100.

14 Compare as well Sasse and Lackner (2018). For the linguistic situation in Belarus’ for example, Hentschel and Kittel (2011a, 2011b) have described that a clear majority of Belarusians state that Belarusian is their native language, although they practically never practise it. A similar situation obviously exists in the Donbas region (cf. Haran et al., 2019).

15 Zeller (2021) offers a sketch of the historical and political background of the linguistic situation in Ukraine, too (cf. as well Hentschel & Taranenko, 2015), which will not be repeated here.

16 This information used to be mentioned in passports and identity cards during Soviet times as “nationality” (Russ. “национальность”, Ukr. “національність”). Note that according to the last Ukrainian census in 2001 (Ukrainian census, 2001) the quantitative relation between Ukrainians and Russian in the three oblasts was roughly seven to one, in the last Soviet census (according to the same source) about six to one. Among our respondents the relation was roughly ten to one. It is not known what the relation is right now. The number of Russians today is definitely not higher than in 2001. It was probably the case that Russians were a little more reluctant to take part in our survey than Ukrainians. Those who refused to take part were of course not asked for their “nationality”.

17 According to the Ukrainian Ukrainian Census (2001) the share of ethnic Russians in the three oblasts was between roughly 20% in Odesa (including the metropole) and roughly 15% in Mykolaïv and Xerson. Presumably it was lower in 2020/2021 due to migration of Ukrainians from the Crimean Peninsula occupied by Russia in 2014 and from the oblasts of Luhans’k and Donec’k where fighting started shortly after. Furthermore, it is safe to assume a certain emigration of Russians to the Russian Federation.

18 As a matter of fact, the term “Suržyk” was not used in the questionnaire. The term is highly stigmatized in Ukrainian society and was therefore avoided (cf. the comprehensive study by Stavyc’ka, 2014). The formulation “mixed language” was always used. The respondents had been informed that this term meant a mixed code with “more of less balanced” shares of Ukrainian and Russian speech. Ukrainian Suržyk is obviously far from having a Russian share that is close to the Ukrainian one, whereas in the similar phenomenon of Belarusian Trasjanka there is a rather balanced relation between Belarusian and Russian, of course with differences in different structural domains such as phonetics/phonology, morphology and lexicon (cf. Hentschel, 2018a, 2018b on this topic). So, hinting at a more or less balanced share of Ukrainian and Russian in the mixed speech was intended to imply that neither a Ukrainian with spontaneous and rather infrequent “ingredients” from Russian was meant, nor a Russian with rather few interferences by Ukrainian (see below in Sect. 3).

19 The statistical analyses were calculated in SPSS 26.

20 Please note that due to the general orientation of the research project, in-depth interviews were only conducted with people who named Suržyk one of their frequently practised codes, mostly in combination with at least one other code, i.e. Ukrainian or Russian. This was different for the closed interviews with 1,200 respondents, which were used for the quantitative analyses. Here the selection was simply a random one.

21 The characteristics of the respondents are given as following: code of respondent; oblast (Od ‘Odesa’ / My ‘Mykolaïv’ / Xe ‘Xerson’); type of place of residence (vi ‘village’ < lv ‘larger village or very small town’ < st ‘small town’ mt ‘medium-sized town’ < lt ‘large town / city’); age; sex (m ‘male’ / f ‘female’); education (s ‘secondary‘ < v ‘vocational’< h ‘higher’; characteristic of the fragment by language). Quotations from the corpus are presented in the original, i.e. in Ukrainian, Russian or the mixed code of Suržyk. By “Ukrainian” / “Russian” we here refer to pure or almost pure codes, some with colloquial elements. The presentation of respondents’ utterances in Suržyk will illustrate only some of the linguistic features that deviate from the Ukrainian (or sometimes Russian) norm, as a linguistic analysis is not at issue in this paper. The English translation conveys mainly the content, linguistic features in the quotations are not emphasized, although the English here is supposed to mirror the colloquial nature of the utterances, such as syntactic elisions, repetitions etc.

22 At least the latter has been frequently reported (cf. e.g. Ažnjuk, 1999; Bacevyč, 2009), though not with special reference to the Black Sea region, traditionally seen as Russian speaking. In Belarus’ Belarusian is as a rule declared as the mother tongue by Belarusians who practically never use it, which is the clear majority of the population (cf. Hentschel and Kittel, 2011a, 2011b).

23 As a matter of fact, asking for one primarily used code in a multilingual or multicodal society may force respondents to a certain overgeneralisation. It is more appropriate to differentiate the choice of codes in the communication with different interlocutors, different as to generation, social position or function, degree of acquaintance etc. (cf. Onuch & Hale, 2018). This will be done for our data in a study to follow.

24 Cf. Del Gaudio (2010, pp. 258–264) for an early hint at such a triglossic constellation.

25 In Figs. 4b and 4c for each respondent and code, a given code was classified as the native language or first language if the respondent evaluated the utterance “X is my mother tongue” or “X is the code I grew up with as a child” as completely correct or rather correct. Note further that the number of respondents included in Figs. 4b and 4c is lower than 1,030, because only those respondents were considered who provided an answer, positive or negative, for all three codes. 140 out of 1,030 did not do so for their native language. (Cf. Fig. 1 regarding these missing answers.) For the first language, 46 respondents did not answer one of the questions or named another language, one of the minority languages on the Black Sea coast. Cf. Hentschel and Zeller (2017) for a comparison with the situation in Central Ukraine.

26 Hentschel and Kittel (2011a, 2011b) report that in Belarus’ such cases are still more widespread, that is, regarding Russian as the language in daily use and Belarusian as the declared native language.

27 Note that we did not ask about competence in Suržyk. An informal pilot survey supported our assumption that for very many people such a question about “competence in Suržyk” would seem to be unanswerable if not outright ridiculous.

28 For an overview of the changing role of the Ukrainian language in society over the last two decades, see Danylevs’ka (2019, pp. 24–40).

29 According to the Law of Ukraine “On the Principles of State Language Policy”, approved in 2012, the use of regional languages, especially Russian, was significantly expanded if the number of speakers of these languages was at least 10% of the population of a region (cf. Bowring, 2014 for more details). The law expired in 2018, according to a ruling by the Constitutional Court. The new law “To ensure the functioning of the Ukrainian language as a state language” was approved by the Verkhovna Rada of Ukraine on 25 April 2019, and took full effect on 16 July of the same year. It establishes the priority of the Ukrainian language in all spheres of public life throughout Ukraine.

30 The empirical basis for their analysis comes from the second research project mentioned above in Footnote 9.

31 Cf. Zeller (2022) in this volume on these points for the Black Sea coast.

32 Points (a) and (g) refer to rather conservative respondents who remain with their “pure” language codes. They are not illustrated with quotations because the in-depth interviews, unlike to the preceding closed interviews, were recorded mainly with Suržyk speakers – see Footnote 21 above.
